# Epithelioid Leiomyoma of the Uterus: A Rare Tumor Mimicking Malignancy

**DOI:** 10.7759/cureus.88675

**Published:** 2025-07-24

**Authors:** Pawni Prabhat, Shravya Kotian, Nupur Kaushik, Akanksha Sharma

**Affiliations:** 1 Pathology, Rama Medical College, Hospital and Research Centre, Hapur, IND

**Keywords:** epithelioid leiomyoma, fibroid, pecoma, stump, uterus

## Abstract

The majority of the smooth muscle tumors of the uterine tract are leiomyomas. A thorough histopathological study of these common lesions is mandatory despite a negligent tendency to omit thorough gross examination. Among the various histopathological subtypes of leiomyomas, epithelioid leiomyoma comprises an uncommon variant that does not display the classic spindle cell morphology of a conventional leiomyoma and could be confused histopathologically with smooth muscle tumor of uncertain malignant potential (STUMP), perivascular epithelioid cell tumor (PEComa), leiomyosarcoma, and endometrial stromal sarcomas (ESS).

In this case report, we present an interesting case of epithelioid leiomyoma in a 38-year-old female patient with complaints of persistent lower abdominal pain for a year. A contrast-enhanced magnetic resonance imaging of the pelvis revealed a well-defined, heterogeneously enhancing mass lesion with a broad base towards the lateral body of the uterus and the lower uterine segment. The findings were suggestive of a subserosal/broad ligament fibroid (FIGO VII) with signs of degeneration. Intraoperatively, the large subserosal fibroid had distorted the normal anatomy, making it challenging to dissect the cervix. Therefore, a subtotal hysterectomy with bilateral salpingo-oophorectomy was done. Histopathological examination and immunohistochemistry led to the diagnosis of epithelioid leiomyoma.

## Introduction

Epithelioid leiomyomas are an extremely rare tumor with few cases reported in the literature. Patients usually present with abnormal bleeding, abdominal pain, menorrhagia, and a mass per abdomen [1]. They bear an epithelioid morphology with cells displaying a round or polygonal appearance, unlike their conventional counterparts, which have a spindled or elongated appearance. They can pose a difficulty with smooth muscle tumor of uncertain malignant potential (STUMP) [2], endometrial stromal sarcoma (ESS), and perivascular epithelioid cell tumor (PEComa) [3], which are aggressive neoplasms that morphologically overlap with the relatively indolent epithelioid leiomyomas. A low mitotic count, lack of significant atypia, and necrosis are often used as pointers towards a leiomyoma [4]. However, due to the rarity of this variant, the overall prognosis of this variant remains largely unknown [5]. Here we report a rare case of epithelioid leiomyoma in a 38-year-old female patient who underwent subtotal hysterectomy with bilateral salpingo-oophorectomy. Due to intraoperative difficulty and the gross appearance of the specimen, clinical differential diagnosis of possible malignancy could not be excluded.

## Case presentation

**Figure 1:**
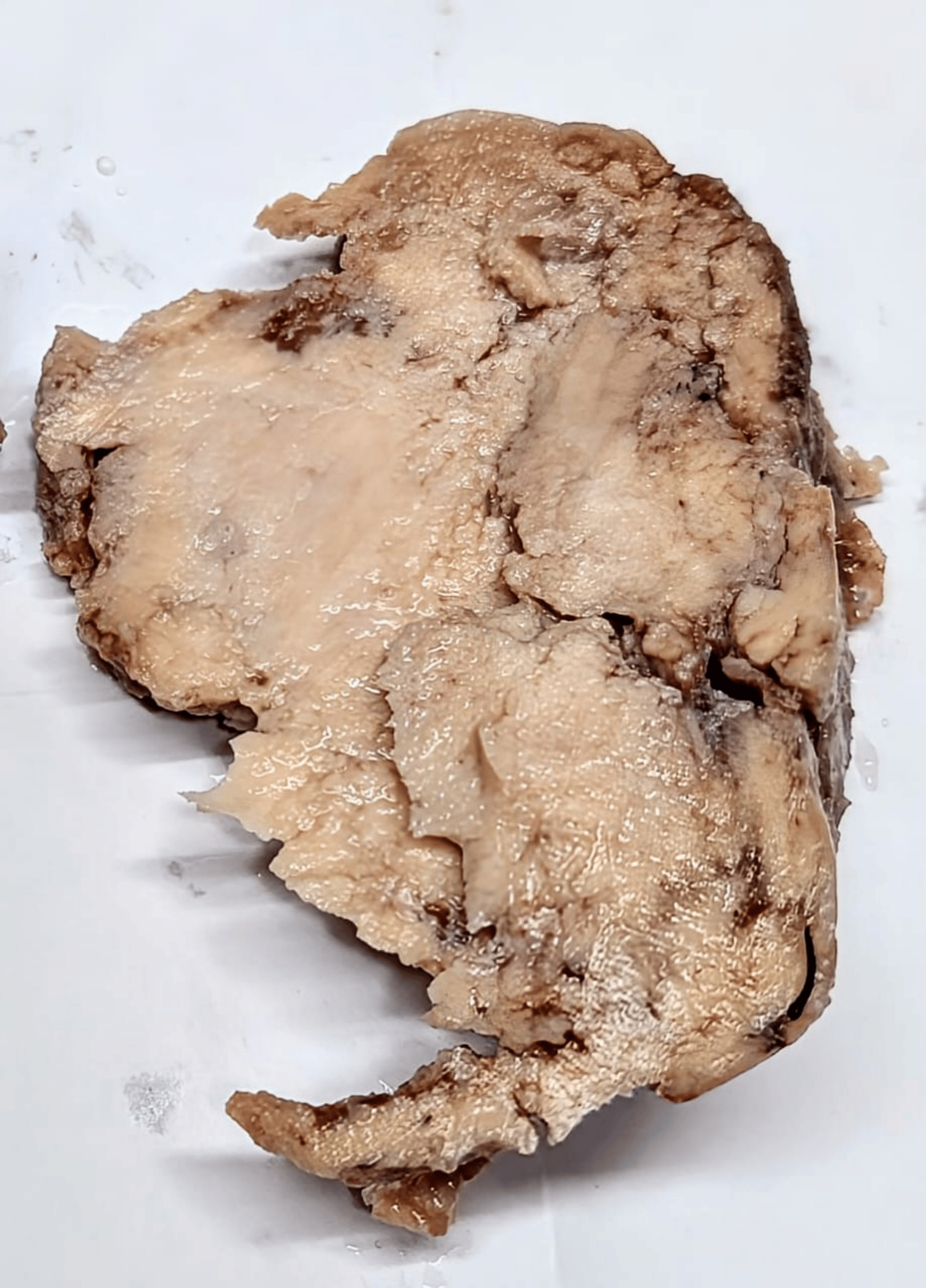
Cut surface of specimen showing solid, homogeneous firm to soft grey-white areas without well-defined whorled appearance

A 38-year-old P3L3 female patient presented with persistent lower abdominal pain for the past one year, which was mild to moderate in intensity and aggravated on micturition. There was no significant history of abnormal uterine bleeding and other co-morbidities except for hypothyroidism. Obstetric history revealed three spontaneous deliveries at term. Her vital signs were stable. Hematological investigations revealed a hemoglobin level of 9.3 g/dl (range 12-15g/dl), and biochemical investigations were within normal limits. Trans-vaginal ultrasound examination (TVS) revealed a bulky anteverted uterus measuring 103x41x40 mm with an endometrial thickness of 8 mm and a homogenous myometrium. A contrast-enhanced magnetic resonance imaging (CE-MRI) of the pelvis was suggested for further evaluation, which revealed a well-defined heterogeneously enhancing mass lesion showing heterogeneous in signal intensity on T2W1, hyper to isointense on T2W1, and iso to hypointense on T1W1. The lesion was described as having a broad base towards the lateral body of the uterus and lower uterine segment. It was abutting the left ovary and urinary bladder with maintained flat planes. These findings were suggestive of a subserosal/broad ligament fibroid (FIGO VII) with signs of degeneration. Uterus and bilateral ovaries were normal in size, shape, and echotexture. Given the patient’s completed family and persistent symptoms, a decision was made to proceed with total abdominal hysterectomy with bilateral salpingectomy. Intraoperatively, the large subserosal fibroid had distorted the normal anatomy, making it challenging to identify and dissect the cervix. Therefore, instead, a subtotal hysterectomy with bilateral salpingo-oophorectomy was done. The specimen was sent to the histopathology department at our institute. On gross examination, the lesion measured 9.5x9x7.5 cm, and it had a well-circumscribed nodular appearance. The cut surface showed solid, homogeneous grey-white areas that were firm to soft in consistency (Figure 1).

**Figure 2:**
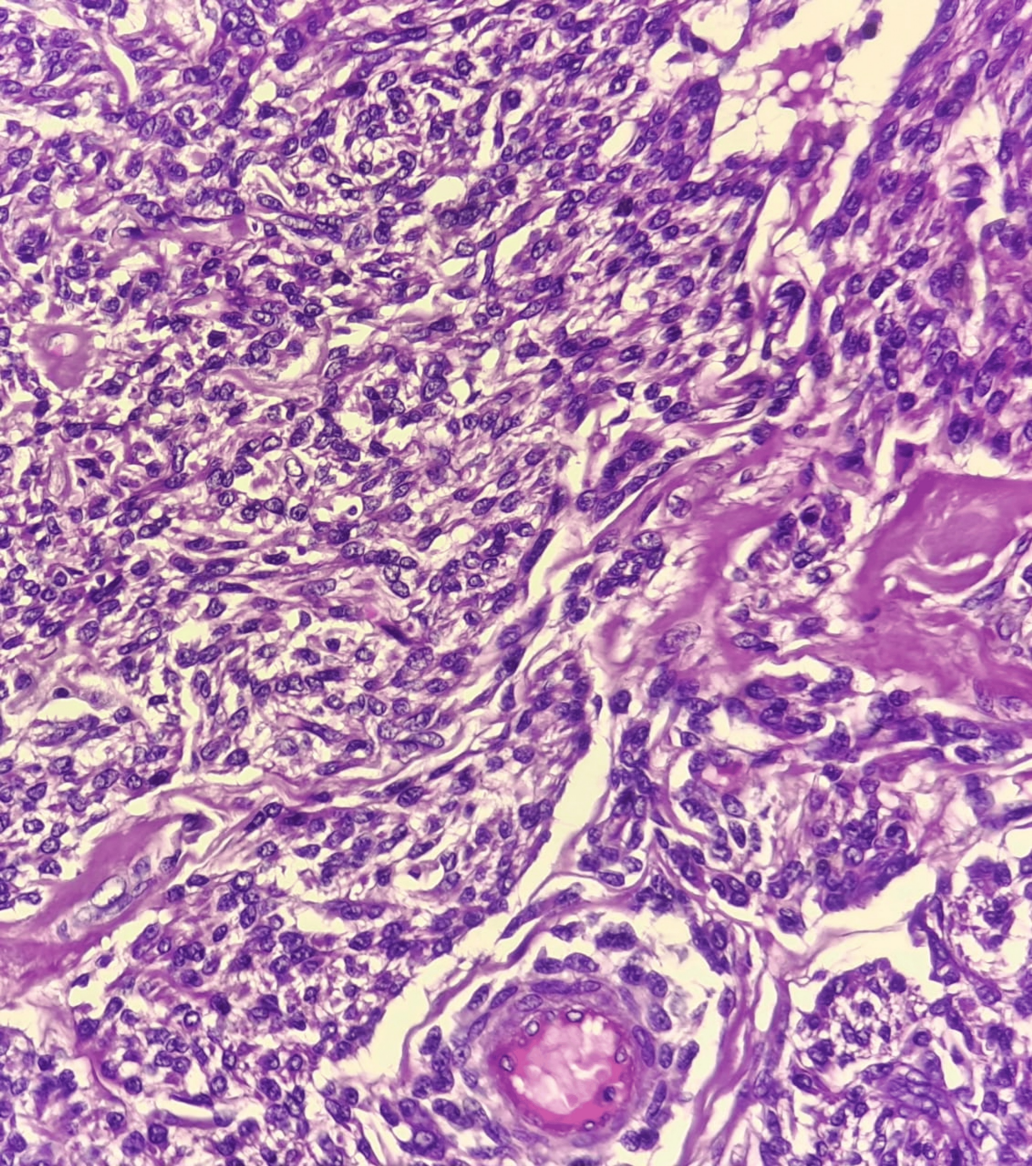
Hematoxylin and eosin (40×) stained section of tumor showing tumor cells arranged in sheets

**Figure 3:**
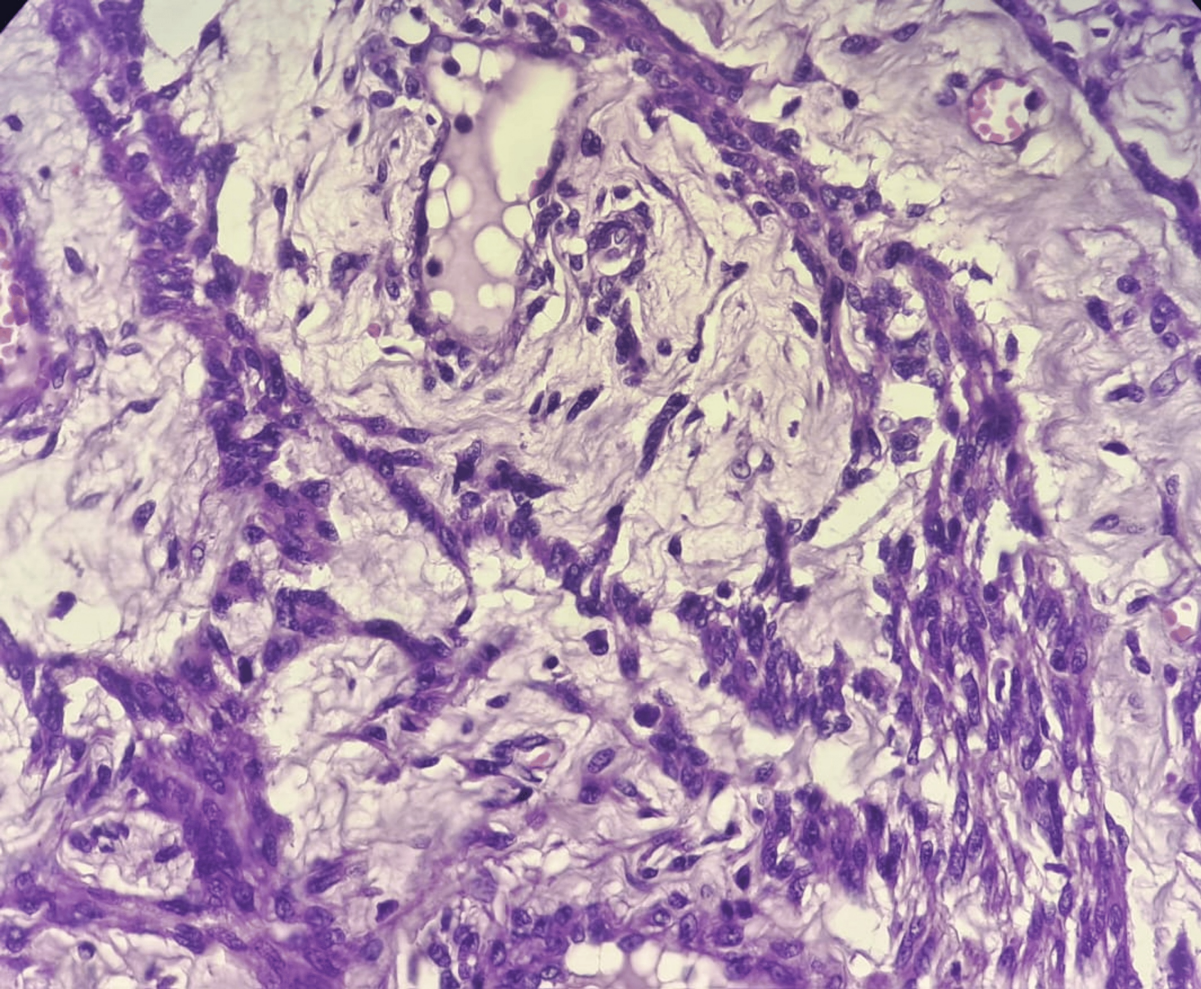
Hematoxylin and eosin (40×) stained sections of the tumor showing tumor cells arranged in cords with cystic and edematous changes

A well-defined whorled appearance was not noted, even with extensive sampling. Hematoxylin and eosin-stained sections from multiple representative sites showed tumor cells arranged in sheets, nests, and cords along with muscular arteries. Individual tumor cells had oval nuclei and eosinophilic to clear cytoplasm. Sheets of polygonal cells, at places showing peritheliomatous arrangement with minimal mitosis, were also seen. Areas of edematous, cystic change, and hyalinization were observed (Figures 2, 3). No significant nuclear atypia or necrosis was observed. The mitotic count was 0-1/10 high-power field (HPF). Due to the unusual gross and microscopic features, a differential diagnosis of STUMP, PEComa, and ESS was also considered, along with epithelioid leiomyoma. Immunohistochemical (IHC) examination with a panel of markers comprising desmin, smooth muscle actin (SMA), HMB-45, CD10, and Ki-67 was used. The polygonal cells showed diffuse positivity for SMA and desmin. HMB-45 and CD10 were negative.

**Figure 4:**
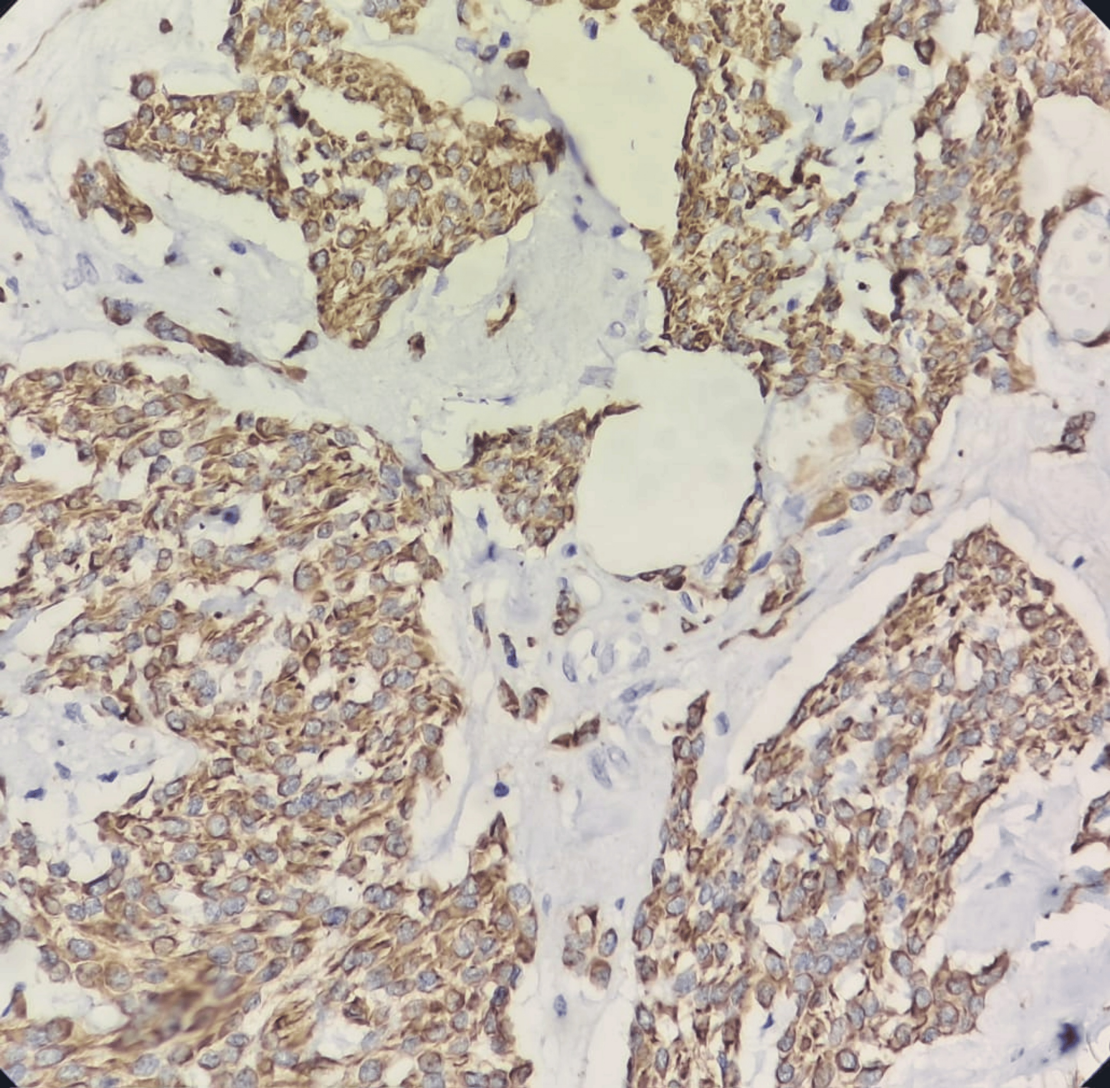
Immunohistochemistry (40×) showing polygonal cells with strong cytoplasmic positivity for desmin

**Figure 5:**
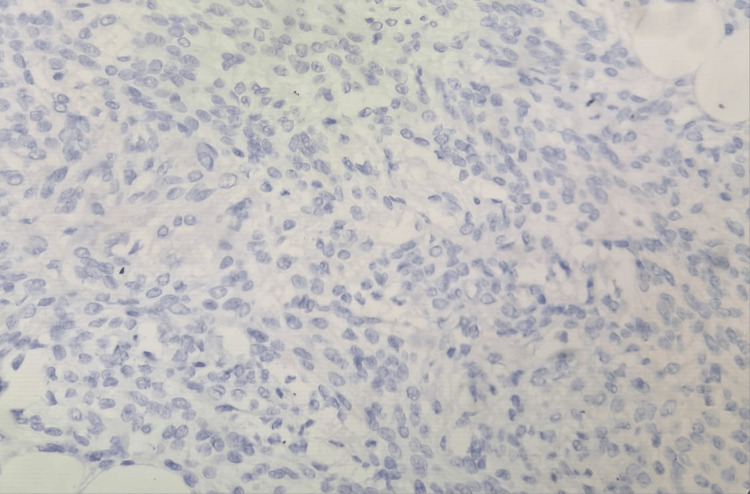
Immunohistochemistry (40×) for HMB-45 is negative

**Figure 6:**
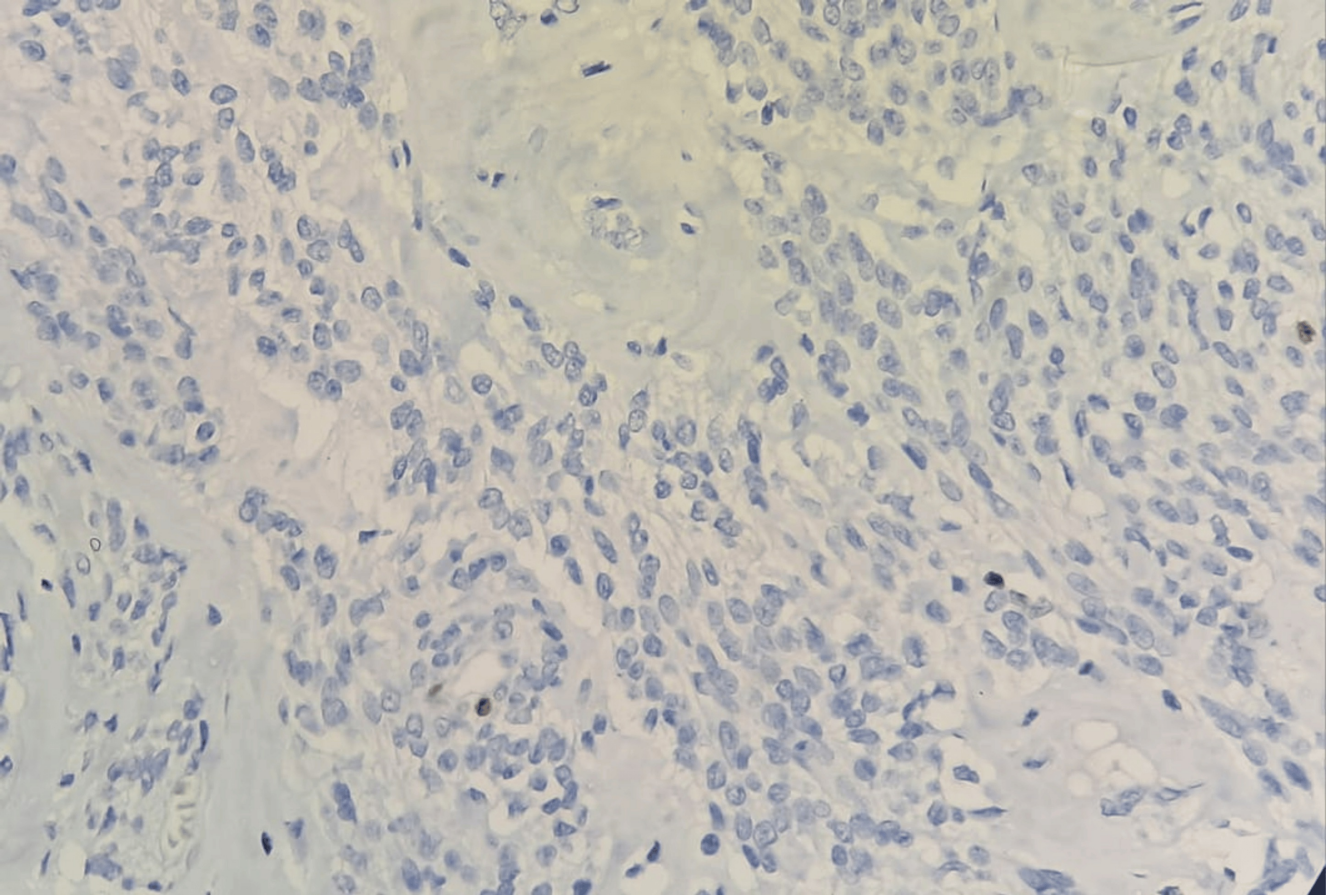
Ki-67 immunohistochemistry (40×) showing nuclear positivity in 1% of tumor cells

Ki-67 proliferation index showed approximately 1-2 % positivity at the hot spot (Figures 4-6). The light microscopy features, in conjunction with immunohistochemical markers, confirmed the diagnosis of epithelioid leiomyoma. Regular follow-up was advised as a variant leiomyoma carries an increased risk of recurrence. The patient was discharged on the seventh postoperative day and was free of any kind of morbidity on two sequential follow-up visits. Post these visits, however, we lost the patient to follow-up.

## Discussion

Leiomyomas are very common benign smooth muscle tumors affecting approximately 70% of women in the reproductive age group and commonly arising from the female reproductive system. Despite its benign and asymptomatic nature, abnormal uterine bleeding (AUB), pelvic heaviness, pain, constipation, increased abdominal girth, infertility, miscarriages, and preterm labor are complications that cause great discomfort [6]. There are several histopathological variants of leiomyomas, like cellular leiomyoma, mitotically active leiomyoma, atypical leiomyoma, and epithelioid leiomyoma [7]. Epithelioid leiomyomas comprise three variants, which are leiomyoblastoma, clear cell leiomyoma, and plexiform leiomyoma; however, mixtures of various patterns are common [8]. This is a rare smooth muscle tumor of the uterus and has not been documented extensively.

In our case, the specimen received in the histopathology department did not possess the classical gross and microscopic appearance of a leiomyoma. Keeping this unusual morphology in mind, a differential diagnosis of STUMP, PEComa, and endometrial stromal sarcoma was also considered, along with epithelioid leiomyoma. After extensive sampling, features such as necrosis, significant nuclear atypia, or increased mitosis were not observed. This, along with a low Ki67 proliferation index, ruled out STUMP. Negative staining for HMB-45 and CD10 ruled out the possibility of PEComa and ESS. Strong positive staining for desmin and SMA favored the diagnosis of epithelioid leiomyoma.

The management is the same for all leiomyomas, despite morphological variations, except for certain variant types. However, for proper classification and patient management, knowledge of tumor variants is crucial, as long-term prognosis and risk of recurrence can vary [9]. The clinical course of epithelioid leiomyoma is unclear due to a lack of studies relating to its prognosis. Because of the increased risk of recurrence, a regular follow-up is needed in patients with at least two of the following findings, which are tumor size more than 6 cm, 2-4 mitotic figures/10 HPF, and moderate to severe cytological atypia and necrosis [6]. In our case, the tumor was 9.5x9x7.5 cm in size, with 0-1 mitosis/10 HPF, and there was no atypia or necrosis. Although molecular advances, such as the identification of MED12 gene mutations, have enhanced our understanding of smooth muscle tumors, many cases are still diagnosed based on histological evaluation [10].

## Conclusions

Epithelioid leiomyoma is a rare variant of leiomyoma. Grossly and microscopically, it can be confused with malignant tumors or tumors of malignant potential. Extensive sampling, careful histopathological examination, supplemented by immunohistochemistry, and knowledge of this uncommon variant are important for prognostication and follow-up. Treatment usually involves surgical excision, and prognosis is favorable, but careful diagnosis is important as they have potential for atypical features with an increased risk of recurrence. After extensive sampling, our case lacked significant atypia and necrosis, mitotic count was 0-1 mitosis/10 HPF; however, the size was more than 6 cm. Though benign, a careful follow-up was still advised because of the rarity of this tumor and the possible risk of recurrence.
